# Advances in IL–2–based cancer immunotherapy and the synergistic role of stem–like CD8^+^ T cells in IL–2 and PD–1 combination therapy

**DOI:** 10.3389/fimmu.2026.1756987

**Published:** 2026-09-17

**Authors:** Huiquan Gao, Shubo Wang, Huiru Yuan, Bingxiu Zhou, Fuxin Zhou

**Affiliations:** 1 Department of Radiotherapy, The Affiliated Yantai Yuhuangding Hospital of Qingdao University, Yantai, China; 2 Department of Gynecology, The Affiliated Weihai Second Municipal Hospital of Qingdao University, Weihai, China; 3 Department of General and Visceral Surgery, Faculty of Medicine, Medical Center - University of Freiburg, Freiburg, Germany

**Keywords:** cancer immunotherapy, CD122 bias, CD25 bias, cytokine engineering, IL–2, IL–2 variants, immunocytokines, prodrugs of IL–2

## Abstract

IL–2 is a pleiotropic cytokine that plays a central role in the activation and proliferation of cytotoxic lymphocytes and regulatory T cells. Since its discovery in 1976 as a T–cell growth factor and its subsequent U.S. FDA approval for metastatic renal cell carcinoma and melanoma, IL–2 has represented a landmark in cancer immunotherapy. However, high–dose (HD) IL–2 therapy is associated with substantial toxicities, including vascular leak syndrome. Recent advances in structural and receptor biology have clarified the molecular mechanisms underlying IL–2’s dual immunostimulatory and immunoregulatory effects. These discoveries have enabled the design of next–generation IL–2 variants (IL–2v) with improved pharmacokinetics, receptor selectivity, and safety profiles. Importantly, PD–1^+^ TCF–1^+^ stem–like CD8^+^ T cells have recently been identified as a key population sustaining long–term antitumor immunity. TCF–1 is a transcription factor associated with a less differentiated, self–renewing state and marks this progenitor–exhausted subset. By contrast, terminally exhausted T cells exhibit a highly differentiated, poorly proliferative state and limited capacity for functional reinvigoration, whereas progenitor–exhausted cells retain stem–like properties, self–renewal capacity, and responsiveness to therapy. Furthermore, these cells respond robustly to IL–2 and are central mediators of the synergy observed between IL–2 signaling and PD–1 blockade. Mechanistically, PD–1 inhibition releases inhibitory constraints, while IL–2 provides a potent STAT5–driven proliferative and differentiation signal that expands the stem–like CD8^+^ T–cell pool and generates functional effector CD8^+^ T cells. This review summarizes IL–2 biology, the limitations of high–dose IL–2 (HD IL–2) therapy, and emerging strategies, including CD25– or CD122–biased IL–2 variants, tumor–targeted IL–2 prodrugs, and IL–2–based combination therapies designed to harness IL–2’s antitumor potential while minimizing systemic toxicity, with particular emphasis on the role of stem–like CD8^+^ T cells as key mediators of therapeutic efficacy.

## 1 Introduction

Interleukin–2 (IL–2) exerts pleiotropic effects on the immune system and was first discovered in 1976 as a “T–cell growth factor” (TCGF), a discovery that rapidly transformed both basic immunology and the emerging field of cancer immunotherapy (1–3). Owing to its potent ability to stimulate the proliferation and activation of cytotoxic lymphocytes, IL–2 became one of the earliest cytokines explored for therapeutic use in cancer (4, 5). In 1992, the U.S. Food and Drug Administration (FDA) approved high–dose (HD) recombinant IL–2 (aldesleukin) for the treatment of metastatic renal cell carcinoma (RCC), based on seven phase II clinical trials involving 255 patients who received high–dose IL–2 (HD IL–2) (600,000–720,000 IU/kg, equivalent to 37–44 µg/kg) every eight hours for up to 14 doses per cycle. The therapy produced an overall objective response rate (ORR) of 14%, including 5% complete response (CR) and 9% partial response (PR), with a median response duration of 19 months among partial responders (6–8). Subsequently, in 1998, HD IL–2 therapy received FDA approval for the treatment of metastatic melanoma (MM), following results from eight clinical trials involving 270 patients. These trials reported a 16% ORR, comprising 6% CR and 10% PR, with a median response duration of 5.9 months for partial responders (9, 10). A substantial proportion of complete responses to HD IL–2 were durable and associated with long–term survival benefits, with median overall survival exceeding 10 years in some cohorts (11).

Despite these remarkable outcomes, the broader clinical application of HD IL–2 has been restricted by its substantial toxicity profile, particularly capillary leak syndrome (CLS), which can affect both responding and nonresponding patients (12). To address the limitations of conventional IL–2 therapy and to selectively enhance antitumor immunity, a range of IL–2–based immunotherapeutic strategies has been developed, many of which are designed to preferentially activate effector CD8^+^ T cells while reducing regulatory T cell (Treg) expansion and systemic toxicity. Among these approaches, combination strategies have gained particular attention. Notably, recent studies have revealed a compelling synergy between IL–2 signaling and PD–1 blockade, particularly by promoting the proliferation and differentiation of stem–like exhausted CD8^+^ T cells (13). This observation has stimulated considerable interest in therapeutically integrating IL–2 pathway modulation with PD–1–directed interventions.

T–cell exhaustion results from persistent antigen exposure during chronic infection or cancer, leading to prolonged immune activation and CD8^+^ T–cell dysfunction. Exhausted CD8^+^ T cells represent a distinct dysfunctional differentiation state that arises during chronic antigen stimulation, and they progressively lose effector functions, exhibit diminished proliferative capacity, and upregulate multiple inhibitory receptors, including PD–1 (14). Although this exhaustion program serves to limit immunopathology under sustained antigenic stimulation, it also compromises effective T–cell–mediated control of tumors and chronic pathogens. Accordingly, reversing or remodeling the exhausted T–cell state has become a major goal of cancer immunotherapy.

While several recent reviews have summarized IL–2–based immunotherapies and engineered IL–2 variants in cancer, including the 2023 review by Muhammad et al. (15) and the 2024 Trends (16) in Cancer review, these articles largely focused on cytokine engineering strategies, pharmacologic optimization, and the broader clinical landscape of IL–2 therapeutics. In contrast, the present review specifically focuses on the emerging role of PD–1^+^ TCF–1^+^ stem–like CD8^+^ T cells as the mechanistic basis underlying the synergy between IL–2 signaling and PD–1 blockade. We discuss recent advances in PD–1–targeted IL–2 variants, receptor–masked IL–2 immunocytokines, and cis–acting IL–2 delivery platforms through this stem–like T–cell framework and consider their implications for exhausted T–cell differentiation and durable antitumor immunity.

## 2 The biology of IL–2 and its receptors

### 2.1 IL–2 biology

IL–2 is a 15.5–16 kDa cytokine composed of a four–*α*–helix bundle that mediates its biological effects through interactions with different forms of the IL–2 receptors (IL–2Rs), including monomeric, dimeric, and trimeric receptors (17–19). The monomeric IL–2R consists solely of the *α*–chain (CD25) and is primarily localized to the cell membrane, although a small fraction is present in soluble form. It binds IL–2 with low affinity (Kd: approximately 10–^8^ M), insufficient to initiate signal transduction (20). Dimeric IL–2Rs consisting of IL–2R*β* (CD122) and IL–2R*γ*_c_ (CD132) exhibit intermediate affinities (Kd: approximately 10–^9^ M) for binding IL–2. Trimeric IL–2Rs composed of the *αβγ*_c_ have high affinity for binding IL–2 (Kd: approximately 10–^11^ M). The affinity of a trimeric IL–2R binding to IL–2 is 10–100 fold higher than a single dimeric IL–2R. More contacts have been observed at IL–2/CD25 interface than the IL–2R*βγ*_c_ in an analysis of IL–2/IL–2R crystal structure (21, 22). This structural asymmetry provides a mechanistic basis for understanding the preferential expansion of regulatory T cells by native IL–2 and directly motivated the development of CD25–deficient or *βγ*_c_–biased IL–2 variants. These engineered IL–2 molecules aim to enhance effector CD8^+^ T–cell and NK–cell responses while minimizing Treg–mediated immunosuppression, thereby improving the therapeutic index of IL–2–based cancer immunotherapies.

The complex of IL–2/IL–2R transduces intracellular signals through three major pathways: Janus kinase (JAK)–signal transducer and activator of transcription (STAT), phosphoinositide 3 kinase (PI3K)–AKT, and mitogen–activated protein kinase (MAPK) (**Figure 1**) (17, 19). Upon IL–2 binding to its *αβγ*_c_ receptor, the cytoplasmic tails of IL-2R*β* and *γ*_c_ come close together. JAK1 (on IL–2R*β*) and JAK3 (on IL–2R*γ*_c_) undergo trans-phosphorylation, leading to phosphorylation of tyrosine residues within the IL–2R*β* cytoplasmic domain, creating docking sites for other signaling proteins, including STAT5, STAT3 and the adaptor protein Shc (5). This launches the intracellular signaling cascade that drives T cell proliferation and survival. Shc recruits the Grb2/SOS complex to activate the Ras–MAPK/ERK pathway, thereby promoting cellular proliferation and survival. (23, 24). Following phosphorylation, the STAT proteins are phosphorylated, dimerized and translocated into the nucleus (**Figure 1**). While IL–2 signaling also engages the MAPK and PI3K pathways in activated effector T cells, STAT5 serves as the principal downstream mediator of IL–2 signaling required for the development and maintenance of regulatory T cells (Tregs).

**Figure 1:**
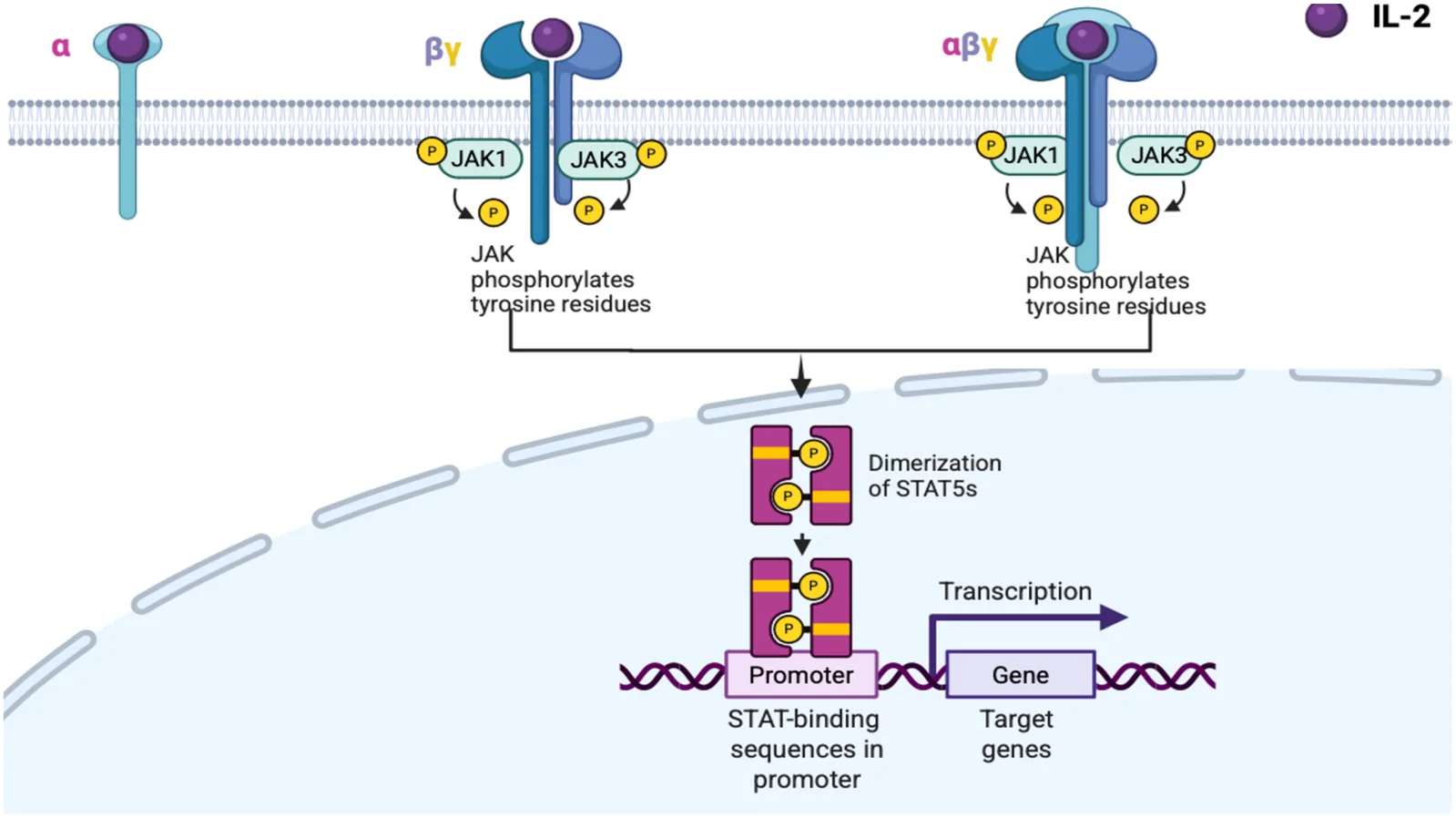
IL–2 receptor complexes, affinity states, and downstream signaling pathways. IL–2 binds receptor assemblies of increasing affinity, including the intermediate–affinity IL–2R*βγ_c_* complex and the high–affinity IL–2R*αβγ*_c_ complex. Upon ligand binding, receptor–associated JAK1 and JAK3 become activated, leading to phosphorylation of receptor tyrosine residues and initiation of three principal downstream signaling cascades: the JAK–STAT5 pathway, the PI3K–AKT–mTOR pathway, and the RAS–RAF–MEK–ERK MAPK pathway. Activated STAT5 dimerizes and translocates to the nucleus, where it regulates transcription of IL–2–responsive genes. Differences in receptor composition determine IL–2 binding affinity, signaling strength, and downstream functional outcomes. Yellow circles labeled “P” indicate phosphorylation events.

Following ligand binding, the IL–2/IL–2R*αβγ*_c_ complex is rapidly internalized, with a half–life of approximately 10–20 min. After internalization, CD25 (IL–2R*α*) is recycled to the cell surface, whereas IL–2, IL–2R*β*, and IL–2R*γ*_c_ are targeted for lysosomal degradation. This degradative process is regulated by proteasome-dependent pathways that influence receptor trafficking. (25–28). For IL–2–dependent T cell proliferation, IL–2R signaling must be sustained for several hours. Some downstream signaling events initiated during IL–2/IL–2R engagement continue to drive cellular responses even after ligand internalization (29). The common *γ* chain is expressed on most hematopoietic cells and is shared by the receptors for IL–4, IL–7, IL–9, IL–15, and IL–21 (23, 30). By contrast, IL–2R*α* is not required for signal transduction but instead increases the affinity of IL–2 for the receptor complex.

During early lymphocyte development, most resting T and B cells do not express the IL–2R*α* subunit until activation. Consistent with this, studies have shown that young IL–2R*α*–deficient mice exhibit normal T and B cell development (31). Upon activation, T cells produce IL–2 and rapidly upregulate IL–2R*α*, leading to the assembly of high-affinity IL–2 receptors. IL–2 signaling activates STAT5, which in turn induces IL–2R*α* expression. As the high–affinity component of the IL–2 receptor, IL–2R*α* enables activated T cells to capture IL–2 more efficiently, thereby strengthening and sustaining IL–2 signaling through a positive feedback mechanism.

### 2.2 Effects of IL–2 on Tregs

IL–2 is crucial for the development, maintenance, and function of Tregs, which maintain self–tolerance and prevent autoimmunity (32). Although Tregs constitutively express high levels of IL–2R*α*, resulting in abundant high–affinity IL–2 receptors, they do not produce IL–2 themselves and therefore depend on IL–2 secreted by activated effector T cells (33). The high-affinity IL–2 receptors enable them to efficiently compete with other T cells for available IL–2 (34). IL–2 contributes to the development of both thymus–derived and peripherally induced Tregs, as well as the maintenance of peripheral Treg homeostasis. Mechanistically, IL–2 signaling activates STAT5, which promotes FOXP3 expression and supports Treg cell function (35). The importance of FOXP3 is underscored by studies showing that its absence in mice results in a complete loss of Tregs and the development of autoreactive T and B lymphocytes (36).

These studies suggest that Tregs respond to IL–2 produced in response to self–antigen stimulation. Consistent with this notion, histocytometric analyses have identified clusters of phospho–STAT5^+^ Treg cells surrounding IL–2–producing CD4^+^ T cells, indicating localized IL–2 signaling within the tissue microenvironment. Further evidence for the importance of this pathway comes from studies in IL–2–depleted mice, where Treg cell numbers remain largely unchanged, whereas the frequency of IL–2–producing CD4^+^ T cells was increased. This finding suggests that IL–2 signaling is required for Treg–mediated suppression of cytokine production by self–reactive CD4^+^ T cells. Together, these observations support a model in which IL–2 participates in a negative feedback loop that limits its own production, either directly through signaling in IL–2–producing CD4^+^ T cells expressing functional IL–2 receptors or indirectly through the activation of suppressive mechanisms in Tregs (37).

In addition, Tregs restrict NK cell activity in the pancreas through an IL–2–dependent mechanism. Treg depletion increased IL–2–responsive transcriptional programs in pancreatic NK cells, whereas HD IL–2 administration overcame Treg–mediated suppression of NK cell function. These findings indicate that Tregs constrain NK cell activation by limiting IL–2 availability, thereby maintaining immune homeostasis (38).

### 2.3 Effects of IL–2 on CD8^+^ T cells

IL–2 plays a central role in the activation, expansion, and functional programming of CD8^+^ T cells and is critical for both the generation of effective cytotoxic responses and the maintenance of long–term immune protection (18). Following antigen recognition, IL–2 promotes the proliferation and differentiation of na¨ıve CD8^+^ T cells into cytotoxic effector cells while also influencing the formation of long–lived memory populations (39). Moreover, IL–2 contributes to the functional reinvigoration of stem–like exhausted CD8^+^ T cells in settings of chronic viral infection and cancer (14). CD8^+^ T–cell differentiation is strongly influenced by the strength and duration of IL–2 signaling, alongside co–stimulatory signals from antigen–presenting cells and cytokines produced by CD4^+^ T helper cells (40–43). High and sustained IL–2 exposure drives strong clonal expansion and terminal effector differentiation, whereas low or transient IL–2 signaling favors the development of memory precursor and exhausted CD8^+^ T cell populations with enhanced persistence and recall responses (44). At the molecular level, IL–2 signaling through the JAK1/3–STAT5 pathway induces transcriptional programs associated with effector function while repressing memory–associated gene expression in a dose–dependent manner, thereby shaping CD8^+^ T cell lineage commitment (39). In addition to regulating differentiation, IL–2 influences the functional quality and durability of CD8^+^ T cell responses. Although excessive or prolonged IL–2 signaling can promote terminal differentiation and contribute to T cell dysfunction during chronic antigen exposure, appropriately regulated IL–2 signals can support the maintenance and reinvigoration of stem–like exhausted CD8^+^ T cells (14). Therefore, IL–2 serves as a key quantitative and qualitative regulator of CD8^+^ T–cell immunity, modulating proliferation, differentiation, and long–term functionality in coordination with antigenic, co–stimulatory, and cytokine–derived signals.

## 3 Limitations of IL–2 based immunotherapies

The inherent drawbacks of IL–2 limit the efficacy and broader application of IL–2–based immunotherapy, including dose–limiting toxicity, preferential activation of Tregs, and poor pharmacokinetic properties such as a short half–life and rapid renal clearance. HD IL–2 monotherapy (600,000–720,000 IU/kg) has been shown to induce complete responses in patients with metastatic renal cell carcinoma and metastatic melanoma. However, such regimens are frequently associated with severe systemic toxicities, including hypotension, CLS, edema, cardiac arrhythmias, and additional hematologic and renal complications (10, 11). To reduce these adverse effects, strategies have focused on modifying IL–2 receptor interactions, either by decreasing receptor affinity to allow higher tolerated doses or by increasing affinity to maintain efficacy at lower doses. Although lower–dose regimens (72,000 U/kg) demonstrate improved tolerability, they generally exhibit reduced therapeutic activity compared with HD intravenous administration (45). Mechanistically, low–dose IL–2 preferentially expands and activates Tregs rather than cytotoxic CD8^+^ T cells because Tregs constitutively express high levels of the high–affinity IL–2 receptor, thereby limiting antitumor immune responses required for therapeutic efficacy. This selectivity contributes to the limitations observed with low–dose IL–2 therapy (46). Consequently, while low doses may impair antitumor efficacy, higher doses are required to stimulate effective antitumor immune responses at the cost of increased systemic toxicity, thereby narrowing the therapeutic index.

Despite recent advances in IL–2–based immunotherapy, several intrinsic properties of IL–2 continue to limit its clinical utility. In addition, IL–2 therapy may activate immunosuppressive populations, such as myeloid-derived suppressor cells (MDSCs), and is also associated with unfavorable pharmacokinetic properties, further limiting current treatment strategies. Collectively, these challenges have driven the development of next–generation IL–2 therapies to enhance receptor selectivity and optimize pharmacokinetic properties.

## 4 Approaches to improve IL–2 based immunotherapies

To overcome the limitations of HD IL–2 therapy, multiple IL–2 engineering strategies have been developed to improve pharmacokinetics, reduce systemic toxicity, and enhance selectivity, including receptor–unbiased, CD25–biased, and CD122–biased formats. Additional efforts focus on restricting systemic IL–2 exposure by selectively delivering IL–2 to tumors. This can be achieved by fusing IL–2 to tumor-targeting antibodies or to inactivation domains connected through protease–cleavable linkers. Among these approaches, CD122–biased IL–2 preferentially activates CD8^+^ T cells while minimizing Treg stimulation, thereby enhancing antitumor immunity within the tumor microenvironment. Representative examples of these engineering strategies and their therapeutic outcomes are discussed below and summarized in **Table 1** and **Table 2**.

**Table 1:** Representative IL-2 cytokines, variants, and prodrug strategies in cancer immunotherapy.

Therapeutic strategy /example	Design feature	Main immunologic effect	Evidence level	Clinical stage/status	Key limitations
High-dose IL-2 (aldesleukin)	Recombinant wild-type IL-2 administered systemically at high doses	Broad activation of CD8^+^ T cells, NK cells, and regulatory T cells owing to nonselective engagement of IL-2 receptors	Peer-reviewed clinical evidence, including long-term follow-up	FDA-approved for selected patients with metastatic melanoma and renal cell carcinoma	Durable responses occur in a small subset of patients, but wider use is limited by severe systemic toxicity, particularly vascular leak syndrome
Bempegaldesleukin (NKTR-214)	PEGylated IL-2 prodrug designed to favor signaling through IL-2R*βγ*_c_	Preferential expansion of effector CD8^+^ T cells and NK cells, with reduced stimulation of CD25^+^ regulatory T cells	Peer-reviewed phase I–III clinical evidence	Phase III development terminated following unsuccessful pivotal trials	Peripheral immune-cell expansion did not translate into sufficient clinical benefit in phase III trials
Nemvaleukin alfa (ALKS 4230)	Engineered IL-2 fusion protein designed to selectively activate intermediate-affinity IL-2R*βγ*_c_ while limiting preferential CD25-mediated regulatory T-cell activation	Expands CD8^+^ T cells and NK cells with comparatively limited expansion of CD25^+^ regulatory T cells	Peer-reviewed early-phase clinical evidence; company-reported phase III results	Phase III development discontinued in platinum-resistant ovarian cancer; development status is indicationdependent	Biological activity has been demonstrated, but the phase III ARTISTRY-7 trial did not establish sufficient clinical benefit in platinum-resistant ovarian cancer
SAR444245 (THOR-707)	Site-specifically PEGylated IL-2 variant engineered to reduce CD25 binding while retaining IL-2R*βγ*_c_ signaling	Expands CD8^+^ T cells and NK cells while reducing high-affinity IL-2 receptor-mediated stimulation of regulatory T cells	Peer-reviewed preclinical evidence and conference-reported early-phase clinical evidence	Investigational; evaluated in phase I/II studies	Early clinical activity has been context-dependent, and the magnitude and durability of clinical benefit remain uncertain
WTX-124	Conditionally activated IL-2 prodrug containing an inactivation domain designed to be removed within the tumor microenvironment	Intended to restrict IL-2 activity to tumors, improve the therapeutic index, and reduce systemic toxicity	Conference-abstract and company-reported phase I/1b evidence	Investigational; phase I/1b	Preliminary activity requires confirmation in larger studies; the optimal dose, therapeutic combinations, and durability of benefit remain under evaluation
BAY 50-4798	CD25-biased IL-2 mutein engineered to favor IL-2R*α* engagement while reducing IL-2R*β* binding	Preferentially stimulates activated T cells but also produces substantial regulatory T-cell activation	Peer-reviewed phase I clinical evidence	Phase I; discontinued	Regulatory T-cell stimulation and limited clinical efficacy reduced its therapeutic value

Evidence note: Evidence level refers to the type and maturity of the supporting data, whereas clinical stage/status indicates the most advanced reported development or regulatory stage. Findings reported only in conference abstracts, interim analyses, or company disclosures should be regarded as preliminary until confirmed in peer-reviewed full-text publications. Clinical development status is current as of July 2026 and may vary by indication.

**Table 2:** Representative tumor-targeted IL-2 immunocytokines and fusion proteins.

Therapeutic strategy /example	Design feature	Main immunologic effect	Evidence level	Clinical stage/status	Key limitations
CEA-IL2v	Carcinoembryonic antigen-targeted antibody fused to an IL-2 variant lacking IL-2R*α* binding	Preferential activation of IL-2R*βγ*_c_-expressing effector cells, with improved localization to CEA-expressing tumors	Peer-reviewed preclinical and early-phase clinical evidence	Investigational; evaluated in early-phase clinical studies	Tumor selectivity and pharmacokinetic properties are attractive, but durable clinical efficacy and optimal combination strategies remain uncertain
PD1–IL2v	Cis–targeted fusion protein designed to engage PD–1 and IL–2 receptors on the same T cell	Selective expansion and differentiation of PD–1^+^ stem–like CD8^+^ T cells with limited systemic IL–2 signaling	Peer–reviewed preclinical evidence	Preclinical/early translational development	The mechanistic rationale is compelling, but clinical efficacy and safety have not yet been established
Hu14.18-IL2	IL–2 fused to an antibody targeting the tumor-associated antigen GD2	Targeted delivery of IL–2 activity to GD2–expressing tumors, with potential enhancement of NK–cell– and T–cell–mediated antitumor immunity	Evaluated in phase I/II studies	Clinical activity has been context–dependent; systemic IL–2–related toxicity and restriction to GD2–expressing tumors remain important limitations	
L19–IL2	IL–2 fused to an antibody fragment targeting the extra-domain B of fibronectin in tumor-associated vasculature	Tumor-directed enrichment of IL-2 activity within the extracellular matrix and localized immune activation	Peer–reviewed preclinical and early-phase clinical evidence	Investigational; evaluated in phase I/II studies	Clinical efficacy varies according to tumor type and combination regimen, and systemic IL-2-related effects may still occur
NHS–IL2LT	Tumor–targeted immunocytokine consisting of IL–2 fused to an antibody that recognizes DNA exposed within necrotic tumor regions	Localization of IL-2 activity within necrotic tumor areas and activation of effector lymphocytes	Peer–reviewed preclinical and early-phase clinical evidence	Investigational; evaluated in phase I/II studies	Clinical activity has been modest or context-dependent, and systemic IL–2–related toxicity remains a potential limitation
IBI363 (TAK-928)	PD–1×IL–2*α*–biased bispecific fusion protein that retains CD25 engagement	Preferential targeting of IL-2 activity to PD-1^+^ tumor-reactive T cells and potential support for the expansion and differentiation of stem-like CD8^+^ T cells	Conference–abstract and company–reported phase I/II clinical evidence	Investigational; global phase III trial initiated	Preliminary efficacy signals require confirmation in randomized phase III trials; long–term efficacy and safety remain under evaluation
PD1-IL2R*α*-IL2	PD-1-targeted, receptor-masked, CD25-retaining IL-2 immunocytokine	Targeted delivery of high-affinity IL-2 signaling to PD-1^+^ tumor-reactive T cells and potential promotion of stem–like CD8^+^ T-cell expansion and differentiation	Peer-reviewed preclinical evidence	Preclinical	The proposed mechanism is promising, but its clinical efficacy, therapeutic window, and safety remain to be established

Evidence note: Preclinical studies, peer-reviewed clinical trials, conference abstracts, and company disclosures represent different levels of evidence and should not be interpreted as having equivalent evidentiary weight. Conference-stage, interim, and company-reported findings are considered preliminary. Clinical development status is current as of July 2026 and may vary by indication.

### 4.1 Unbiased IL–2 formulations

#### 4.1.1 PEG–IL–2

The idea of conjugating PEG chains to IL–2 for an extended circulating half–life originated in 1987, launching nearly a decade of research and clinical trials exploring PEGylated interleukin–2 (PEG–IL–2) for cancer immunotherapy. PEG–IL–2 was first reported to retain its biological function and control tumor growth compared to recombinant IL–2 (rIL–2) (47). Moreover, in mouse models, PEG–IL–2 was shown to induce tumor regression with similar efficacy to HD IL–2 (48, 49), and reduce tumor growth compared to PBS–treated controls in L10 tumor–bearing mice (50). Based on these promising results in preclinical studies, PEG–IL–2 subsequently entered clinical trials. In a phase I clinical trial involving sixty–six patients conducted over a 19–month period, PEG–IL–2 was administered intravenously once weekly via a 15–minute infusion. The maximum tolerated dose (MTD) was established at 20 × 10^6^ IU/m². The toxicity profile was comparable to that of rIL–2, while pharmacokinetic analyses demonstrated a 10– to 20–fold extension of serum half–life relative to the unconjugated cytokine (51).

In another phase I study, weekly intravenous bolus administration of PEG–IL–2 yielded an MTD of 12 × 10^6^ IU/m^2^, again with toxicities similar to those of unconjugated IL–2. A twice–weekly dosing schedule, however, was less well tolerated. Subsequently, 124 patients with metastatic melanoma or renal cell carcinoma (RCC) were randomized to receive either standard HD IL–2 monotherapy or a combination regimen consisting of HD IL–2 followed by PEG–IL–2 maintenance therapy. No treatment–related mortality was observed in each group, and the inclusion of PEG–IL–2 significantly reduced the requirement for intensive care unit support. Overall response rates (including complete and partial responses) for IL–2 monotherapy were 19% in RCC and 15% in melanoma, whereas the combination therapy with PEG–IL–2 yielded 17% and 11%, respectively. Although the addition of PEG–IL–2 did not significantly enhance antitumor efficacy relative to HD IL–2 alone, the combined regimen was well tolerated and demonstrated meaningful clinical activity in both RCC and melanoma (52).

##### 4.1.1.1 Antibody–cytokine fusions or immunocytokines

Another innovative strategy to improve IL–2 therapy is targeted cytokine delivery through antibody–cytokine fusion proteins, also known as immunocytokines. By genetically fusing IL–2 to antibodies or antibody fragments that recognize tumor–associated antigens (TAAs), these constructs enable preferential accumulation of IL–2 at tumor sites while minimizing systemic exposure (53, 54). For example, Hu14.18–IL–2 incorporates an anti–GD2 antibody, enabling selective accumulation of the fusion protein at GD2–expressing tumors and increasing local IL–2 concentrations. In addition, L19–IL–2 is a diabody targeting the extra–domain B (ED–B) of fibronectin, with two human IL–2 molecules fused to the C–termini of the single–chain variable fragments (scFvs). Preclinical studies demonstrated promising antitumor activity *in vivo* (55–57), which subsequently led to phase I clinical evaluation of both Hu14.18–IL–2 and L19–IL–2, where mild and reversible toxicities were observed (58, 59). Moreover, treatment with Hu14.18–IL–2 resulted in stable disease in 58% of melanoma patients following the first treatment cycle. L19–IL–2 subsequently entered phase II clinical trials in combination with dacarbazine for metastatic melanoma (NCT02076646 and NCT01253096).

These studies demonstrated that antibody–mediated delivery could improve the therapeutic index of IL–2 by increasing tumor localization while maintaining an acceptable safety profile. However, the clinical benefits observed so far have been relatively modest, reflecting several inherent limitations of immunocytokines, including heterogeneous TAA expression, restricted penetration into solid tumors due to their large molecular size, and residual systemic exposure. These challenges highlight the difficulty of achieving precise spatial control of IL–2 activity and have stimulated the development of alternative approaches aimed at restricting cytokine activity more selectively within the tumor microenvironment (TME). Among these, prodrug strategies have emerged as a promising platform for improving the safety and efficacy of IL–2–based immunotherapy.

##### 4.1.1.2 Prodrugs

Prodrug strategies have emerged as a promising approach for improving the therapeutic index of cytokine therapies by preserving antitumor activity while minimizing systemic toxicities. A key principle underlying these approaches is the transient masking of cytokine activity, which can subsequently be restored in response to tumor–associated cues. One particularly attractive strategy exploits proteases enriched within the tumor microenvironment to achieve tumor–selective cytokine activation (60). To this end, diverse masking moieties, including PEG chains, constitutively associated cytokine receptors, antibody fragments, and peptides, have been employed to temporarily shield cytokine activity until activation at the tumor site (60, 61).

Bempegaldesleukin (BEMPEG; NKTR–214) is a first–generation engineered IL–2 prodrug that employs a releasable PEGylation strategy to extend the half–life and confer selectivity for the dimeric IL–2R*β/γ_c_*receptor (61). In preclinical models, BEMPEG showed enhanced antitumor activity and significantly improved intra–tumoral CD8/Treg ratio compared to rIL–2, suggesting potential synergy with checkpoint inhibitors (62). These findings were supported by the early–phase clinical trials. In the PIVOT–02 study, the combination of BEMPEG and nivolumab yielded an ORR of 53% and a CR of 34% in metastatic melanoma patients (63). However, these encouraging results did not translate into clinical benefit in large–scale randomized Phase III trials. In PIVOT–09 for renal cell carcinoma, the BEMPEG combination achieved an ORR of 23.0%, underperforming compared to 30.6% observed in the tyrosine kinase inhibitor control arm. Similarly, in the PIVOT IO–001 trial in metastatic melanoma, the addition of BEMPEG to nivolumab resulted in a lower ORR (27.7%) compared to nivolumab monotherapy (36.0%) and nearly doubled the incidence of Grade 3/4 treatment–related adverse events (64, 65).The failure of BEMPEG highlights critical challenges in IL–2 development, including the potential for “overestimation” in early non–randomized cohorts and the limitations of peripheral immune expansion as a surrogate for intra–tumoral efficacy (66, 67). This high–profile discontinuation has necessitated a strategic re–evaluation of other *βγ_c_*–biased agonists, emphasizing the need for more stable engineering and rigorous early–stage comparative trials.

In addition, the Fu group has developed a series of cognate receptor–masked IL–2 prodrugs (68, 69). Among these, WTX–124 comprises wild–type human IL–2 linked to a Fab antibody fragment that inactivates the cytokine, together with a single–domain antibody targeting human albumin to extend its half–life. WTX–124 is currently being evaluated in Phase I clinical trial (NCT05479812). This design aims to enhance immunotherapeutic efficacy by selectively activating immune cells within the TME, while simultaneously minimizing systemic toxicity through tumor–specific cytokine release.

Collectively, the clinical experience with BEMPEG and receptor–masked prodrugs such as WTX–124 highlights both the potential and limitations of prodrug strategies. While these approaches successfully improve the therapeutic index by extending half–life, restricting systemic exposure, and promoting intratumoral effector T cell expansion, they rely primarily on the tumor–penetration property and tumor–associated protease activity, which may not fully translate into durable antitumor responses. These observations underscore the importance of receptor expression patterns in determining IL–2 activity and suggest that more precise engagement of specific immune subsets could further refine efficacy and safety. In this context, CD25–biased IL–2 formulations represent an innovative strategy, exploiting the differential expression of the high–affinity IL–2 receptor to preferentially target regulatory and activated T cells, thereby enabling more selective modulation of the immune response and offering a complementary approach to the strategies discussed above.

### 4.2 CD25–biased IL–2 formulations

An early hypothesis proposed that these adverse effects were primarily mediated by interferon–*γ*_c_–secreting NK cells. Since NK cells were initially believed to express little or no CD25 under resting conditions, researchers pursued IL–2 mutational strategies to bias receptor binding toward the *α*–chain (CD25) (70–73). This led to the development of the first CD25–biased IL–2 mutein, BAY 50–4798, which incorporated an N88R mutation designed to reduce its affinity for CD122 (70, 72). However, results from a phase I clinical trial in patients with RCC and melanoma were disappointing, showing low response rates and prompting discontinuation of further development (73). While BAY 50–4798 selectivity recently activated effector T cells over NK cells, it also preferentially expanded CD4^+^CD25^+^FoxP3^+^Tregs expressing the high–affinity IL–2 receptor *α* chain, suppressing antitumor immunity. The development of neutralizing antidrug antibodies against the N88R mutation likely further restricted its clinical efficacy.

To enhance tumor selectivity and improve the therapeutic index of CD25–biased IL–2, immunocytokines were subsequently developed to target tumor–associated components. Examples include NHS–IL–2LT, which binds DNA–histone complexes, and KY1043, which targets programmed death–ligand 1 (PD–L1) expressed on tumor cells. NHS–IL–2LT retained near–normal biological activity in T–cell lines expressing the high–affinity IL–2 receptor but showed minimal activity in cells expressing only the intermediate–affinity receptor. Compared with control antibody–IL–2 fusion proteins containing wild–type IL–2, NHS–IL–2LT maintained antitumor activity against established neuroblastoma and non–small cell lung cancer metastases in syngeneic mouse models, while exhibiting improved tolerability in immunocompetent mice and cynomolgus monkeys (74, 75). Despite these encouraging preclinical findings, a phase I clinical trial involving 48 patients failed to demonstrate convincing efficacy, with no complete or partial responses observed (76). Building on these findings, Innovent developed an anti–PD–1/IL–2 fusion protein (IBI363), designed to simultaneously block PD–1 and deliver a cis–targeted CD25–biased IL–2 signal to reinvigorate exhausted TSTs. A phase I trial demonstrated that IBI363 was well tolerated and showed preliminary antitumor activity in patients with advanced non–small cell lung cancer (NSCLC) and melanoma (NCT05290597; NCT05460767) (77).

More recently, KY1043 demonstrated robust antitumor activity in MC38 tumor models. Interestingly, KY1043 preferentially expanded TCF–1^+^PD–1^+^ precursor–exhausted CD8^+^ T cells in murine models (5), a subset associated with favorable responses to immunotherapy. Although substantial Treg expansion was observed in peripheral tissues, this phenomenon was not evident within the tumor microenvironment, resulting in an increased CD8^+^/Treg ratio in tumors but a decreased ratio in the periphery. These findings suggest that peripheral Treg expansion may not compromise IL–2–mediated tumor control and may instead mitigate systemic toxicities, including capillary leak syndrome (CLS), a major dose–limiting toxicity of IL–2 therapy.

### 4.3 CD122–biased IL–2 formulations

Another major engineering strategy has focused on preferential engagement of the IL–2R*βγ*_c_ complex in order to enhance effector responses while limiting dependence on CD25–mediated signaling. SAR444245 (THOR–707) is a PEG–IL–2 fusion protein developed by Synthorx, featuring a single, permanently attached PEG chain linked via a novel amino acid insertion site. This specific PEG placement prevents IL–2 from binding to the IL–2R*α* subunit, thereby biasing its activity toward CD8^+^T cells and NK cells that express the intermediate–affinity IL–2R*βγ_c_* complex (78).

In preclinical models, SAR444245 exhibited a prolonged half–life and successfully stimulated CD8^+^ T and NK cell expansion without inducing CLS or Treg activation typically associated with rhIL–2 (79). While the agent inhibited tumor growth in melanoma and colon cancer models, it failed to achieve complete tumor regression as a monotherapy (80). However, the therapeutic potential was enhanced when used in combination with PD–1 inhibitors, resulting in a 36% complete response rate in CT–26 tumor–bearing mice. In early clinical evaluation, SAR444245 was administered once every three weeks and demonstrated a manageable safety profile with no dose–limiting toxicities, effectively expanding peripheral CD8^+^ T and NK cells without inducing CLS or significant IL–5 elevation (81). However, the clinical trajectory of the agent underscores the complex challenge of translating this robust immunological expansion into meaningful antitumor efficacy, as overall results in advanced solid tumors fell below projected benchmarks (82). Consequently, Sanofi halted the initial phase II trials in October 2022 to pursue schedule intensification. However, despite robust peripheral immune activation, clinical activity in patients with advanced solid tumors was modest and did not translate into the degree of antitumor efficacy anticipated from preclinical studies (82). In contrast to these early–stage pipeline transitions, another clinically advanced example of a CD122–biased IL–2 strategy is nemvaleukin alfa (ALKS 4230), an engineered IL–2 variant designed to selectively activate the intermediate–affinity IL-2R*βγ_c_*. In the phase I/II ARTISTRY–1 trial, updated results published in 2024 demonstrated a manageable safety profile and modest antitumor activity in patients with advanced solid tumors, both as monotherapy and in combination with pembrolizumab. The combination cohort achieved an objective response rate of 13%, including complete and partial responses, indicating biological activity while highlighting the need for further optimization of IL–2–based therapeutic strategies (83).

In addition, Roche developed an engineered IL–2 variant (IL–2v) that is fused to antibodies targeting tumor–associated components. This IL–2v retains affinity for the IL–2R*βγ_c_* complex while abolishing binding to the IL–2R*α* subunit. For example, CEA–IL–2v and FAP–IL–2v demonstrated enhanced tumor selectivity and improved pharmacokinetic properties compared with wild–type IL–2 (84);?. Preclinical studies have shown that both CEA–IL–2v and FAP–IL–2v can synergize with PD–L1 checkpoint inhibitors and antibody–dependent cellular cytotoxicity (ADCC)–competent antibodies to elicit potent antitumor activity. In a phase I clinical trial, CEA–IL–2v was evaluated in combination with atezolizumab for the treatment of locally advanced or metastatic solid tumors (NCT02350673). Meanwhile, a phase Ib/II study assessed FAP–IL–2v in combination with other agents for metastatic pancreatic ductal adenocarcinoma (NCT03193190).

More recently, Roche reported the development of PD–1–IL–2v, which enables cis engagement of PD–1 and IL–2R*βγ_c_* on the same cell. This design promotes the differentiation of stem–like CD8^+^ T cells into effectors without engaging CD25, leading to improved therapeutic efficacy (85). PD–1–IL–2v is currently being investigated in a phase I clinical trial (NCT04303858). In parallel, research by Innovent Biologics Inc. revealed that IL–2R*α*–biased agonists, compared with IL–2R*βγ_c_*–biased variants, more effectively expand tumor–specific CD8^+^ T cells (TSTs), thereby exhibiting superior antitumor efficacy. Recent work further suggests that CD25–engaging, PD–1–targeted IL–2 designs may better direct cytokine activity toward tumor–reactive T–cell populations. Notably, PD–1^+^CD25^+^CD8^+^ tumor–infiltrating lymphocytes are enriched for tumor–specific T cells, supporting the rationale for PD–1–targeted IL–2 strategies that retain IL–2R*α* engagement (69).

### 4.4 Lessons from IL–2 evolution: from systemic bias to structural precision

The clinical evolution of engineered IL–2 variants highlights a progressive shift from simple pharmacokinetic optimization toward increasingly precise, cell type–specific cytokine delivery. The disappointing clinical performance of first–generation IL–2 engineering strategies, exemplified by bempegaldesleukin, demonstrated that static masking of CD25 binding through PEGylation alone is insufficient to achieve meaningful clinical benefit. Although PEGylation extended the circulating half–life, reduced renal clearance, and mitigated severe toxicities such as capillary leak syndrome, it did not provide selective delivery to the TME, resulting in widespread systemic cytokine exposure. Under conditions of chronic antigen stimulation, intratumoral effector T cells progressively acquire features of exhaustion. Consequently, systemic IL–2 delivery may fail to provide the localized signaling intensity and spatial restriction required for efficient reinvigoration of tumor–reactive T cells.

In contrast, PD–1–targeted cis–delivery approaches, exemplified by IBI363, represent a promising strategy to overcome these spatial limitations. By tethering the cytokine payload to an anti–PD–1 backbone, these constructs preferentially target TCF–1^+^PD–1^+^ stem–like CD8^+^ T cells within the tumor microenvironment. Localized cis–delivery provides coordinated checkpoint blockade and cytokine stimulation, thereby promoting the differentiation of stem–like progenitors into effector progeny capable of mediating durable antitumor responses.

### 4.5 Comparative perspective on IL–2 engineering strategies

Collectively, the diverse IL–2 engineering strategies reflect attempts to balance efficacy, safety, and pharmacokinetics. PEGylation and Fc–fusion approaches primarily aim to improve pharmacokinetics by extending circulating half–life and reducing dosing frequency. However, these strategies generally do not fully address the issue of Treg activation. Immunocytokines and prodrug strategies attempt to improve the therapeutic index by restricting IL–2 activity to the tumor microenvironment, thereby limiting systemic exposure and toxicity. Clinical experience with agents such as bempegaldesleukin highlights the difficulty of translating robust peripheral immune activation into durable antitumor responses.

Receptor–biased IL–2 variants, particularly CD122–biased agonists, preferentially stimulate effector CD8^+^ T cells and NK cells while minimizing activation of high–affinity CD25^+^ Tregs, making this a promising approach. Nevertheless, these strategies face challenges related to manufacturability, pharmacokinetic optimization, and demonstrating clear clinical superiority over existing immunotherapies. Emerging evidence suggests that the most promising future strategy is likely to combine receptor–biased or tumor–targeted IL–2 variants with immune checkpoint blockade, such as PD–1 inhibitors, to selectively expand stem–like CD8^+^ T cells while limiting systemic toxicity.

Collectively, the evolution of IL-2 engineering highlights several recurring principles. Strategies focused primarily on improving pharmacokinetics or promoting broad peripheral immune expansion, including PEGylated formulations and some CD122–biased agonists, have often failed to translate promising preclinical outcomes into durable clinical benefit. Tumor-targeted prodrugs and immunocytokines improve the therapeutic index by restricting cytokine activity, but their efficacy remains dependent on tumor–specific activation and tissue penetration. Emerging evidence suggests that therapeutic success may depend less on simple receptor bias and more on precise spatially and cellularly restricted delivery of IL-2 signals to PD-1^+^TCF-1^+^ stem-like CD8^+^ T cells. Thus, the evolution of IL-2 therapies reflects a shift from systemic cytokine exposure toward biologically informed, cell-selective cytokine delivery.

## 5 Advances in IL–2 and PD–1 combination therapy

### 5.1 The exhaustion of CD8^+^ T cells

T-cell exhaustion refers to the loss of memory and effector functions in T cells during chronic infection or cancer, driven by persistent antigen exposure or inflammatory signals from pathogens or tumor cells (86). The exhausted CD8^+^ T cells were first identified in 1998 by using a mouse model of chronic lymphocytic choriomeningitis virus (LCMV) infection, in which virus–specific CD8^+^ T cells are exposed to chronic viral antigens (87, 88). Exhaustion of T cells has also been observed following chronic antigenic stimulation in nonhuman primates and humans (89–91). Moreover, cytokine dysregulation is a key feature of the exhausted T–cell phenotype. During T–cell exhaustion, the production of IL–2, interferon–*γ* (IFN–*γ*), and tumor necrosis factor–*α* (TNF–*α*) progressively declines. However, T–cell exhaustion does not imply a complete loss of function. Exhausted T cells retain the ability to produce specific effector molecules, including inflammatory cytokines and granzymes, which can contribute to tumor cell killing. (86, 92). Exhausted T cells display sustained high expression of inhibitory receptors (IRs), including PD–1, T–cell immunoglobulin and mucin domain 3 (TIM–3), lymphocyte activation gene 3 (LAG–3), CTLA–4, and T–cell immunoreceptor with immunoglobulin and ITIM domains (TIGIT). They also showed altered cytokine production and distinct epigenetic, transcriptional, and metabolic profiles (86, 91, 93). Thus, T–cell exhaustion represents an adaptive response to chronic antigenic stimulation and serves to limit tissue damage caused by excessive or prolonged immune activation (86, 94).

In addition, exhausted CD8^+^ T cells are now understood to comprise functionally distinct subpopulations rather than a uniform dysfunctional state (91, 95). In particular, PD–1^+^ TCF–1^+^ stem–like CD8^+^ T cells retain self–renewal capacity and the potential to differentiate into cytotoxic effector–like progeny, and they are thought to represent the principal population responsive to PD–1 blockade (16, 95). By contrast, terminally exhausted CD8^+^ T cells display more severe functional impairment, limited proliferative capacity, and reduced responsiveness to reinvigoration (16, 95). This distinction is especially important for understanding the synergy between PD–1 blockade and IL–2, because these therapies appear to act primarily by expanding and redirecting the stem–like progenitor pool rather than by rescuing terminally exhausted cells (16).

### 5.2 PD–1–directed IL–2 combination therapy

Several studies have established PD–1 as a central regulator of T–cell exhaustion, demonstrating that removing this inhibitory “brake” restores CD8^+^ T cell function and reduces viral burden (96). This foundational insight provided the mechanistic rationale for PD–1–directed immunotherapies, which have transformed the prognosis within multiple cancer types for patients. More recent work has revealed that PD–1^+^ TCF–1^+^ stem–like CD8^+^ T cells are not committed to the exhausted fate and that their differentiation trajectory can be reshaped by IL–2 signaling. The ability of IL–2 to expand these stem–like CD8^+^ T cells, which maintain self–renewal and the ability to differentiate into cytotoxic CD8^+^ T cells, underlies a key immunological basis for combining IL–2 pathway modulation with PD–1 blockade.

For example, Deak and colleagues reported that PD–1–IL–2v, a PD–1-targeted immunocytokine designed to engage PD–1 and IL–2R*βγ_c_* on the same stem–like CD8^+^ T cell, effectively promoted the expansion of the pool of stem–like CD8^+^ T cells while generating highly functional effector CD8^+^ T cells in both chronic infection and tumor models. In preclinical studies, treatment with PD–1–IL–2v led to pronounced tumor control and durable survival benefits, outperforming PD–1 blockade alone or IL–2v delivered in trans via a FAP–targeted construct. Notably, PD–1–IL–2v achieved complete tumor regression in a substantial proportion of treated animals, whereas the combination of parental PD–1 antibody with FAP–IL–2v produced only limited long–term responses (85). Moreover, Fusi and colleagues reported consistent findings using a PD–1–targeted IL–2v engineered to abolish CD25 binding, further supporting the therapeutic potential of PD–1–directed IL–2 variants (97).

The PD–1/IL–2*α*–biased bispecific fusion protein IBI363 has shown preliminary clinical activity. According to recent conference proceedings and interim registry updates (98), IBI363 demonstrated promising ORR in patients with immunotherapy–refractory advanced acral and mucosal melanoma. However, as these data originate primarily from early–phase, single–arm cohort expansions presented in abstract form, large–scale, peer–reviewed randomized trials are required to firmly establish the therapeutic advantage over established checkpoint-based regimens.

In addition to PD–1–IL–2 fusion strategies, combining PD–1 blockade with IL–2 has also produced promising synergistic outcomes. Hashimoto and colleagues demonstrated that combining PD–1 blockade with wild–type IL–2 modulates the differentiation trajectory of PD–1^+^TCF–1^+^ stem–like CD8^+^ T cells. This regimen generated transcriptionally and epigenetically distinct effector CD8^+^ T cells that closely resembled the highly functional effectors observed during acute viral infection—an outcome not achieved with PD–1 monotherapy. These emerging effector cells expressed elevated levels of the high–affinity IL–2 receptors, highlighting the importance of CD25 engagement in mediating the synergy between IL–2 and PD–1 inhibition. Indeed, blocking CD25 or using an IL–2 variant that abrogates CD25 binding largely abolished the synergistic effects. These mechanistic insights underscore the therapeutic potential of PD–1 and IL–2 combination strategies and provide a strong foundation for ongoing translational efforts (13).

Together, these advances illustrate the rapidly expanding landscape of IL–2–based strategies designed to enhance CD8^+^ T–cell immunity while minimizing systemic toxicity, providing a strong rationale for the continued clinical development of PD–1–directed IL–2 therapies.

### 5.3 Unresolved controversies and the paradox of receptor bias

The rapid evolution of engineered IL–2 formulations has shifted the therapeutic paradigm from high–dose, non–selective cytokine administration toward targeted molecular design. However, clinical transitions have exposed critical gaps between robust preclinical immune expansion and actual clinical benefit. Resolving these mechanistic discrepancies requires a critical examination of the central controversies that shape the future of IL–2–based combination regimens.

#### 5.3.1 The paradox of complete CD25 abrogation in checkpoint inhibitor combinations

Next-generation IL–2 engineering has traditionally aimed to abolish CD25 binding to limit systemic Treg expansion and mitigate CLS. However, recent translational and clinical evidence indicates that complete CD25 abrogation may be counterproductive when combined with PD–1 blockade. While CD25-abolishing variants avoid Treg stimulation, they also lose high-affinity engagement of PD–1^+^ TCF–1^+^ stem-like CD8^+^ T cells, which drive the proliferative burst and sustain durable responses during PD–1 therapy.

Codarri Deak et al. demonstrated that an anti–PD–1 antibody fused with a CD25-silent IL–2 variant could be optimized through avidity-driven cis-targeting. By fusing the IL–2 variant directly to the anti–PD–1 backbone (PD–1–IL2v), the molecule forces physical proximity to PD–1^+^ TCF–1^+^ stem-like CD8^+^ T cells, rescuing proliferation and differentiation into highly functional effector cells without expanding peripheral Tregs or inducing CLS (85).

However, Overacre–Delgoffe et al. (2023) showed that robust antitumor responses and preservation of a less-exhausted T-cell phenotype require direct engagement of the trimeric high-affinity IL–2R*αβγ_c_* complex on tumor-specific effector CD8^+^ T cells. Agonists retaining CD25 binding, when combined with PD–1 blockade, significantly enhanced tumor-reactive T-cell expansion and maintained their less-exhausted state (99).

These findings highlight that complete CD25 abrogation can limit signaling in tumor-specific effector lineages, whereas cis-targeting can partially compensate for this loss by leveraging spatial proximity. The optimal therapeutic strategy may lie in context-dependent CD25 engagement, such as PD–1–directed, receptor-masked, or cis-acting designs that localize high-affinity IL–2 signaling to PD–1^+^ tumor-infiltrating CD8^+^ T cells while minimizing systemic exposure. Such approaches preserve the potent differentiation cues necessary for durable antitumor immunity.

### 5.4 The mechanism of the synergistic combination therapy of PD–1 blockade and IL–2

Recent insights into CD8^+^ T cell status and their lineage relationships have greatly improved our understanding of how PD–1 blockade enhances CD8^+^ T cell responses in cancer and chronic infection. PD–1 therapy acts differently across the heterogeneous CD8^+^ T cell compartment. It promotes the proliferation and differentiation of PD–1^+^ TCF–1^+^ stem–like CD8^+^ T cells, which reside in lymphoid tissues such as tumor draining lymph nodes (TDLNs) and tumor associated tertiary lymphoid structures (TLSs). The expansion of this population generates a greater pool of transitory effector–like CD8^+^ T cells, which then enter the circulation and migrate to tumor or infection sites. Consistent with this, the presence of TLSs and progenitor exhausted T cells (Tpex) within the TME correlates with responsiveness to checkpoint blockade (95, 100, 101), and an increase in proliferating PD–1^+^ CD8^+^ T cells has been observed in the peripheral blood of patients following PD–1 blockade therapy (102, 103). In addition, PD–1/PD–L1 pathway inhibition at the tumor or infection site restores the effector functions of transitory T cells and exhausted stem-like CD8^+^ T cells, thereby facilitating the elimination of malignant or infected cells (104–106). However, the terminally exhausted CD8^+^ T cells remain unresponsive to PD–1 blockade, IL–2 alone, or the combination therapy (16). Collectively, PD–1^+^TCF–1^+^ stem–like CD8^+^ T cells are not irrevocably committed to the exhaustion pathway, and IL–2 signaling can redirect their fate toward a more functional effector state, highlighting IL–2 as a crucial driver of effector differentiation in the setting of persistent antigen (16) (**Figure 2**).

**Figure 2:**
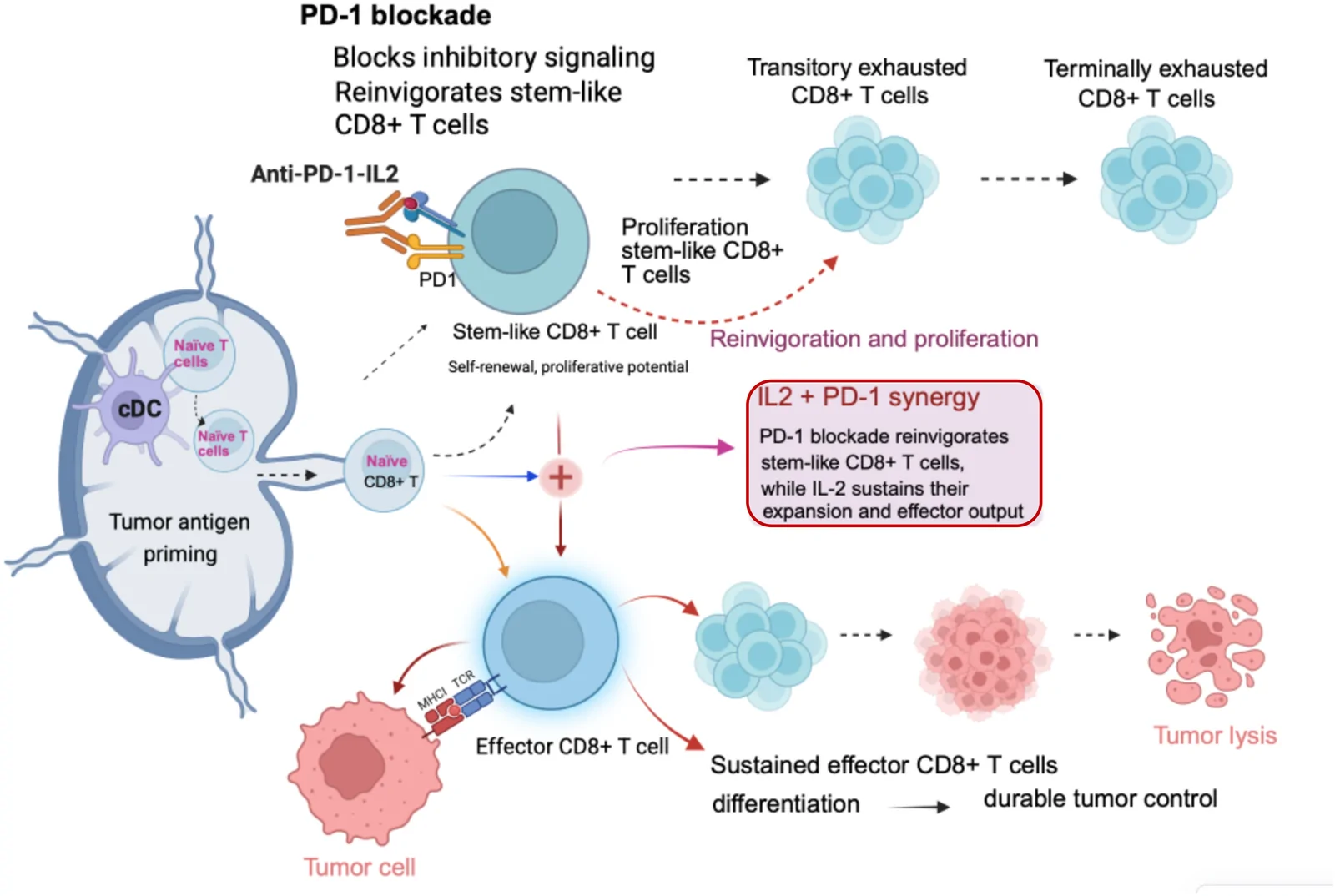
Conceptual model of the synergistic effects of stem-like CD8^+^ T cells in IL–2 signaling and PD–1 blockade. Tumor antigens are presented by conventional dendritic cells (cDCs) to na¨ıve CD8^+^ T cells, contributing to the generation of stem–like exhausted CD8^+^ T cells with self–renewal and proliferative capacity. PD–1 blockade may alleviate inhibitory signaling and reinvigorate these stem–like cells, promoting their proliferation and differentiation into transitory and terminally exhausted CD8^+^ T–cell populations. Targeted delivery of IL–2 through fusion to an anti–PD–1 antibody may further support the expansion, survival, and effector potential of PD–1^+^ stem–like CD8^+^ T cells. Based on emerging preclinical and clinical evidence, the coordinated effects of PD–1 blockade and IL–2 signaling may help maintain the stem–like CD8^+^ T–cell pool, promote continuous generation of effector cells, and enhance antitumor activity, thereby potentially contributing to sustained tumor control and tumor–cell lysis. This figure presents a conceptual model, and the magnitude and durability of these proposed effects may vary depending on the therapeutic construct, tumor type, and immunological context.

From a therapeutic perspective, the delineation of distinct CD8^+^ T–cell exhaustion states provides actionable opportunities for intervention instead of merely descriptive classification. The identification of PD–1^+^ TCF–1^+^ stem–like CD8^+^ T cells as a progenitor population suggests that effective immunotherapies should prioritize the expansion and differentiation of this subset rather than attempting to reinvigorate terminally exhausted cells. This insight has direct implications for therapeutic design, supporting strategies that deliver proliferative and differentiation signals, such as IL–2 or IL–2 variants, in a spatially and temporally coordinated manner with immune checkpoint blockade. Moreover, several features of the exhausted CD8^+^ T–cell compartment may serve as candidate predictive biomarkers to guide patient stratification, identify individuals most likely to benefit from PD–1– and IL–2–based combination therapies, and monitor treatment response. In particular, the abundance of PD–1^+^TCF–1^+^ stem–like CD8^+^ T cells may indicate whether a tumor contains the progenitor pool required for productive responses to checkpoint blockade and IL–2–driven differentiation. Likewise, expression of IL–2 receptor components, especially CD25, may help determine which T–cell populations are likely to respond to high–affinity IL–2 signaling. Additional features, such as tertiary lymphoid structures, transcriptional signatures associated with progenitor versus terminal exhaustion states, and the spatial organization of immune subsets within the tumor microenvironment, may further refine patient stratification. Although these features remain candidate rather than validated clinical biomarkers, they provide a useful framework for future biomarker–guided immunotherapy. Collectively, leveraging the functional heterogeneity within exhausted CD8^+^ T–cell populations enables a shift from nonspecific immune reinvigoration toward more precise, state–directed immunotherapeutic interventions.

## 6 Conclusion and future directions

In summary, while IL–2 remains a cornerstone cytokine in cancer immunotherapy, its clinical utility has been constrained by severe toxicities and limited efficacy in only a subset of patients. Advances in structural biology, protein engineering, and receptor biology have paved the way for next–generation IL–2 variants with enhanced selectivity, improved pharmacokinetics, and more favorable safety profiles. Future directions include the development of conditionally activated and tumor–targeted IL–2 formulations, receptor-biased variants, and fusion proteins that enable localized or cell-specific cytokine delivery while minimizing systemic exposure. In addition, rational combination strategies integrating these optimized IL–2 molecules with immune checkpoint inhibitors, adoptive cell therapies, and CAR–T approaches will likely be essential for maximizing therapeutic benefit. Collectively, these innovations hold promise for more fully realizing the antitumor potential of IL–2, particularly in rationally designed and combination–based therapeutic regimens, while minimizing toxicity and broadening its clinical impact across diverse cancer types.

Emerging evidence suggests that the identification of PD–1^+^ TCF–1^+^ stem–like CD8^+^ T cells as the principal cellular substrate underlying the synergy between IL–2 signaling and PD–1 blockade represents a conceptual shift in cancer immunotherapy. Rather than broadly reinvigorating exhausted T cells, future therapeutic strategies should prioritize the selective expansion, maintenance, and differentiation of this progenitor population. IL–2–based interventions, particularly engineered variants that enable controlled, cell–restricted, or temporally coordinated signaling, are well positioned to complement PD–1 blockade by driving productive effector differentiation from stem–like CD8^+^ T cells. Furthermore, integrating IL–2 pathway modulation with checkpoint inhibition, biomarker–guided patient stratification, and rational dosing schedules may further enhance therapeutic durability while minimizing toxicity. Collectively, leveraging stem–like CD8^+^ T cells as both therapeutic targets and candidate biomarkers offers a promising framework for the next generation of combination immunotherapies.
